# Scaffolding low quality genomes using orthologous protein sequences

**DOI:** 10.1093/bioinformatics/bts661

**Published:** 2012-11-18

**Authors:** Yang I. Li, Richard R. Copley

**Affiliations:** Wellcome Trust Centre for Human Genetics, University of Oxford, Oxford, OX3 7BN, UK

## Abstract

**Motivation:** The ready availability of next-generation sequencing has led to a situation where it is easy to produce very fragmentary genome assemblies. We present a pipeline, SWiPS (Scaffolding With Protein Sequences), that uses orthologous proteins to improve low quality genome assemblies. The protein sequences are used as guides to scaffold existing contigs, while simultaneously allowing the gene structure to be predicted by homology.

**Results:** To perform, SWiPS does not depend on a high N50 or whole proteins being encoded on a single contig. We tested our algorithm on simulated next-generation data from *Ciona intestinalis*, real next-generation data from *Drosophila melanogaster*, a complex genome assembly of *Homo sapiens* and the low coverage Sanger sequence assembly of *Callorhinchus milii*. The improvements in N50 are of the order of ∼20% for the *C.intestinalis* and *H.sapiens* assemblies, which is significant, considering the large size of intergenic regions in these eukaryotes. Using the CEGMA pipeline to assess the gene space represented in the genome assemblies, the number of genes retrieved increased by >110% for *C.milii* and from 20 to 40% for *C.intestinalis*. The scaffold error rates are low: 85–90% of scaffolds are fully correct, and >95% of local contig joins are correct.

**Availability:** SWiPS is available freely for download at http://www.well.ox.ac.uk/∼yli142/swips.html.

**Contact:** yang.li@well.ox.ac.uk or copley@well.ox.ac.uk

## 1 INTRODUCTION

New sequencing technologies have lowered the cost of generating genomic DNA sequence to the point where high coverage of a moderately sized animal genome (e.g. <1 Gb) can be obtained for a few thousand euros. Currently, short read lengths (i.e. 100–250 bp) present challenges for assembling sequence into a complete genome, usually owing to an inability of short reads to bridge repetitive sequence. This problem can be circumvented by using paired end or mate paired reads with different insert sizes long enough to span repetitive regions. For instance, the genome of the giant panda (Li *et al.*, 2010a) and, more recently, the one of the naked mole-rat (Kim *et al.*, 2011) have been sequenced using short read sequencing technology alone and achieved high quality. Both projects used multiple libraries; the latter used 18 paired-end sequencing libraries with 8 different insert sizes spanning 170 bp to 20 kbp. This strategy has the disadvantage of incurring significant extra cost, and large insert size libraries require larger amounts of DNA that may not be readily available for some taxa.

Apart from mainstream *de novo* assemblers, several groups have worked on incorporating available related assembled genomes into the assembly process (Pop and Salzberg, 2008). For instance, it is standard procedure (e.g. the orangutan genome project, Locke *et al.*, 2011) to use whole genome alignments to build super scaffolds by aligning the new assembly to the high quality genome of closely related species. Many species, however, are sequenced precisely because they are not closely related to species for which a complete genome is available.

Thus, other approaches have been adopted to increase assembly contiguity. In Mortazavi *et al.* (2010), for example, researchers exploited the eukaryote exon–intron gene structure and used RNA-seq data to scaffold contigs from a *Caenorhabditis* nematode genome, increasing the initial N50 from 5.0 to 9.4 kb. This strategy obviously requires the generation of RNA-Seq data in addition to genomic sequence, and genes that are not expressed in the RNA-Seq library will not be available for scaffolding.

As protein sequences are generally well conserved across distant taxa (compared with non-coding or DNA sequences), a possible way to deal with fragmented initial assemblies is to use orthologous proteins to guide the scaffolding of contigs that encode fragments of the same proteins. Surget-Groba and Montoya-Burgos used orthologous proteins to guide transcriptome assembly by mapping contigs onto a related protein set and improved the N50 of their zebrafish transcriptome assembly by up to 42% (Surget-Groba and Montoya-Burgos, 2010). The presence of introns, large intergenic regions and the much larger volume of data involved in genome assemblies, however, make this a fundamentally more difficult problem. Salzberg *et al.* introduced a ‘gene-boosting’ algorithm that used amino acid sequences from predicted proteins to improve the scaffolding of a 6 Mb bacterial genome, although again, as bacterial proteins lack introns, this is a somewhat easier problem to approach (Salzberg *et al.*, 2008).

Here, we present an algorithm that uses proteome sets from different species to guide scaffolding of genome assemblies of various qualities (including highly fragmented ones, e.g. N50 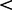 3 kb). In this article, we use the term scaffolding to refer to the process of ordering and orienting contigs, although we recognize that we do not estimate distances between contigs. We call this SWiPS (Scaffolding With Protein Sequences). Existing algorithms and strategies, including the Ensembl genebuild process for low-coverage assemblies, and GPIPE (Heger and Ponting, 2007), are not suited for highly fragmented assemblies where a gene may be spread over multiple contigs. SWiPS uses orthologous proteins (possibly from distantly related species) as guides to link exons of the same protein that may be situated on different contigs. To our knowledge, only one other published method, ESPRIT (Dessimoz *et al.*, 2011), does this. ESPRIT was used to bridge 666 *Callorhinchus milii* contigs from a Sanger-based assembly together and reported precision of ∼80%. ESPRIT, however, assumes a uniform evolutionary rate over the entire length of split proteins, something that is clearly questionable in the general case.

We present a novel score optimization framework for scaffolding contigs and a strategy to optimize this score to improve the contiguity of novel assemblies, reduce their fragmentation, as well as predict the content of their exomes. We tested SWiPS on a set of genomic assemblies of simulated *Ciona intestinalis* next-generation sequencing data, real sequencing data from *Drosophila melanogaster*, as well as the Sanger sequenced, but low coverage (1.4×) assembly of *C.milii* and a pre-assembled genome of *Homo sapiens*.

## 2 METHODS

### 2.1 Outline of method

**Fig. 1.**
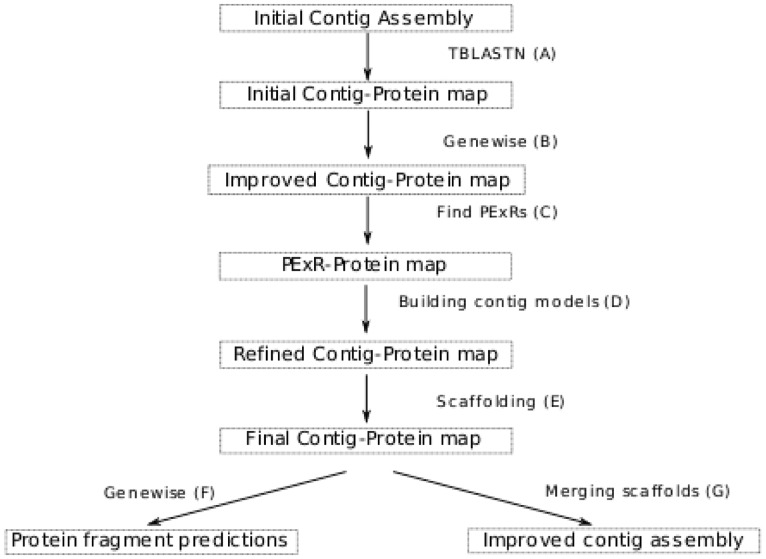
(**A**) Proteins in the chosen guide proteome are mapped to contigs in the initial assembly with tblastn. All overlapping mappings considered to be significantly worse than the best one are removed. (**B**) Proteins with good mappings on contigs are re-aligned with genewise to obtain single nucleotide resolution of exon predictions. (**C**) Putative Exonic regions (PExRs) are predicted by overlapping contig regions containing multiple homologous protein fragments. (**D**) PExRs within each contig are used to establish a refined contig–protein orthology model, only allowing contigs to map to different proteins if the associated sets of PExRs show no overlap. Then, contig gene models are built using this mapping. (**E**) A score optimization heuristic is used to scaffold contigs together according to the total score of protein models. (**F**) genewise is used once again on the protein scaffolds to predict protein fragments. (**G**) A graph theoretic approach is used to find the local order of the contigs and their strand

The overall method is outlined in Figure 1 and described in detail in this section. In brief, we first identify contigs that include protein-coding exons. Next, we order and orient these contigs by mapping them to proteins, allowing multiple contigs to map to the same protein. We compute similarity scores between proteins and contigs according to a distance matrix. We use these scores to scaffold contigs together greedily by iteratively picking the best scaffold model and removing those contigs that match it. Lastly, we link scaffolds into super-scaffolds by examining contigs with multiple proteins which, in turn, are assigned to multiple contigs.

### 2.2 Determining coding contigs and exonic regions

To determine which contigs might be protein coding, proteins from user-specified guide proteomes are mapped onto the set of all contigs using tblastn (Gertz *et al.*, 2006). An initial protein–contig mapping is obtained by assigning a contig to a protein if it (i) has a mapping e-value below 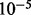 and (ii) that e-value is under five orders of magnitude less significant than the most significant contig mapping to the same protein region. Proteins are allowed to map to multiple contigs. Two contigs are considered to map to the same protein region if the regions they map to overlap by 15 amino acids. These parameters are chosen to be relaxed compared with similar usage in previous studies, e.g. (Hahn *et al.*, 2007). However, more conservative parameters can be chosen at the cost of sensitivity to gain running speed.

We refine the tblastn-based protein to contig mapping using genewise, to more precisely define exon boundaries (contigs of sufficient length will contain multiple exons from the same gene). We align the template protein to the contig using the ‘623’ model of genewise (Birney *et al.*, 2004), successively searching the regions of contig flanking each alignment until no more high scoring regions are found (although this is in principle possible with the looping models of genewise, in practice these yield results that are hard to interpret). The results from all iterations are then merged, and all mapping coordinates are corrected to yield one or multiple protein–contig pairwise alignments.

After re-aligning with genewise, a mapping from contig intervals to protein intervals is established. Then, for every contig, the longest contiguous regions with at least one protein mapping to every base in each region are found and defined to be Putative Exonic Regions (PExRs), i.e. PExRs are intervals on contigs for which every base is covered by at least one protein mapping. This definition of PExR is useful even if we expect a single orthologous protein region to be homologous and map to the entirety of a PExR. We found that the mapping of orthologous proteins suffered from edge effects, i.e. orthologous regions with little similarity can only be aligned if they are flanked by orthologous sequences showing high similarity. Because of the lack of potential flanking region with high similarity at the edges of orthologous regions, there is a drop in sensitivity in the detection at the edges of these regions. Furthermore, splice variants can have exons with alternative 3′ and 5′ splice sites. By combining all protein mappings of the same contig region, we were able to predict regions coding for exons with higher specificity.

### 2.3 Scaffolding contigs by optimization of overall protein to contig mappings

At this stage, we have a refined mapping of proteins to contigs. Using these mappings, we compute a similarity score for each protein–contig mapping by summing the bit scores across the aligned regions defined by genewise according to a distance matrix (BLOSUM62 in our case). We now must order the contigs into scaffolds, by defining orthology relationships between the template protein and contigs mapping to different regions of that protein. In cases where there is a clear one-to-one orthologous relationship between the guide protein and its cognate in the newly sequenced genome, this task is conceptually straightforward. Our method, however, must allow us to disentangle orthology relationships within gene families using fragmentary sequences. This complicates the procedure considerably.

The protein–contig similarity scores only give us a partial picture of the orthology relationships between proteins and contigs. The distribution of similarity scores is strongly correlated with their lengths, and the strength of stabilizing selection can be highly variable across loci. Therefore, orthology relationships between a particular protein and contig cannot be called without considering all other mappings between the contig and other proteins. In order to take into consideration all mappings at once, we posed the problem of scaffolding contigs as an optimization problem for which we maximize the similarity scores between contigs and guide proteins without creating inconsistencies. That is, we assign contig gene models, i.e. regions of a contig that are predicted to encode the exons of a single gene, to protein regions so that (i) each pair of contig gene model and protein region is the coupling most likely to be orthologous (according to similarity scores), (ii) there are no two contig gene models associated to the same protein regions and (iii) we use as few proteins as possible as scaffold models. The reasons for (i) and (ii) should be fairly obvious, as we can only scaffold contigs together (and extend protein gene models) if they contain PExRs orthologous to different regions of the same protein. Importantly, it can be seen that the implementation of condition (i) in SWiPS is analogous to the best reciprocal hits approach to identifying orthologous proteins. Condition (iii) is necessary for resolving the problem of contigs mapping to legitimate orthologs from different proteomes. By using as few protein scaffolds as possible, we can reduce the spread of contig mappings over multiple protein orthologs or splice variants. In SWiPS, the implementation of these ideas is summarized by the definition of two concepts, the scaffolding power and the maximal scaffoldable set, which are described next.

### 2.4 Scaffolding power and maximal scaffoldable set

**Fig. 2.**
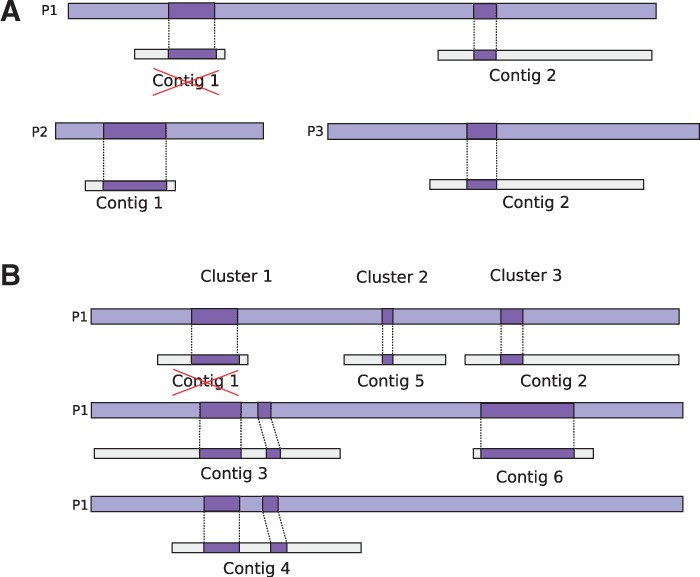
(**A**) Although contig 1 and 2 are mapped to protein P1, they have better mappings on protein P2 and P3, respectively. The similarity score between contig 1 and P1 is lower than α = 0.75 times the similarity score between contig 1 and P2, and is removed from P1. The similarity score between contig 2 and P1 is within α of the similarity score between contig 2 and P3, and both mappings are kept. (**B**) Contigs with similarity to the P1 are clustered according to the region of similarity. Here, contigs 3 and 4 are clustered together in contig cluster 1, contig 5 is the only contig in contig cluster 2, and contig 2 and 6 are clustered in contig cluster 3

**Fig. 3.**
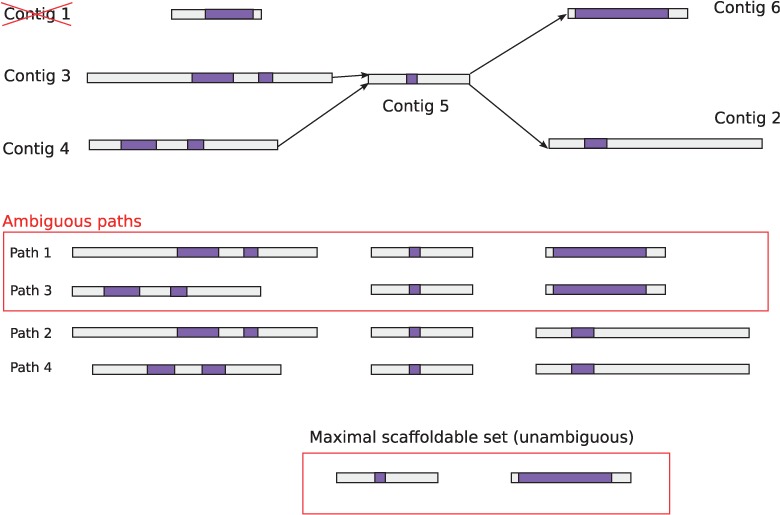
The scores for all possible combinations of contigs in each region are computed (including combinations with no contigs from clusters), and the lowest scoring paths are removed. The higher scoring paths (with at least β times the highest scoring path) are then compared; contigs present in the highest scoring (ambiguous) paths are kept and determine the maximal scaffoldable set

For each potential guide protein, we retrieve all refined contig mappings and define regions of the guide protein into which the contigs cluster (Fig. 2). To allow for mapping edge effects, contigs cluster together if they overlap by 15 nucleotides or more. In all contig clusters, contigs that have a low similarity score are removed if they are lower than α times the highest similarity score (default α = 0.75) (e.g. contig 1 in Fig. 3). The mappings of multiple contigs to the guide protein can then be viewed as a graph (Fig. 3). We calculate the score of all possible paths through this graph, by exhaustively combining all contigs from different regions, summing the individual contig–protein match scores to get an overall score for each path. All paths having scores with at least β (default β = 0.5) times the highest scoring path are then compared, and contigs present in all higher scoring paths are kept and form a maximal scaffoldable set, with a score referred to as the scaffolding power. The default values of α = 0.75 and β = 0.5 were chosen to be conservative, and make SWiPS less likely to produce chimeric sequences from different paralogs within the target genome. These two parameters, and how they affect the performance of SWiPS in the presence of paralogs, are discussed next. However, we have tested several values for α and β on our *C.intestinalis* runs and found that, for both, increased values resulted in increased numbers of scaffolded contigs (and N50), but also in increased rates of scaffolding error.

Contigs encoding in-paralogs, i.e. genes with many-to-one or many-to-many relationships with members of the guide proteins, cannot be reliably scaffolded because they result from one or more duplication events subsequent to the divergence from genes in the guide proteome. Contigs resulting from duplication events prior to the divergence from genes in the guide proteome (out-paralogs), however, can be scaffolded. In SWiPS, α is used to uncouple contig–protein pairings that have lower similarity scores than the one with the highest score (e.g. contig 1 and P1 in Fig. 2). For example, an α = 1 forces every contig to be coupled unambiguously with the protein with which it shares the highest similarity score, whereas an α = 0 implies that all contig–protein pairings are kept for subsequent steps. Consequently, a high α can be used when a complete set of closely related proteins is used as guide proteome, and contigs encoding out-paralogs will be coupled unambiguously with their orthologous proteins. However, if the set of closely related proteins is incomplete, a high α may result in erroneous contig–protein couplings that are considered unambiguous, thereby leading to scaffolding errors. Our choice of 0.75 as the default value for α is thus conservative and allows the use of incomplete protein sets.

Although α takes incomplete protein sets into consideration, our maximal scaffoldable set approach (which relies on β) takes into account contigs encoding in-paralogs that cannot be scaffolded. If two contigs encode in-paralogous sequences (contig 3 and 4 in Fig. 2B and 3), they will form distinct paths that have similar scaffolding powers (e.g. path 1 and 3 in Fig. 3). Therefore, they will be excluded from the maximal scaffoldable set (Fig. 3). Because the number of in-paralogs are potentially high, we chose β to be conservative.

Once the scaffolding power of each protein is computed, the protein scaffold with the highest scaffolding power is iteratively picked, and the maximal scaffoldable sets (and scaffolding power) of all other proteins are updated so that a contig gene model (the contig region) is only used once. Picking the protein scaffold with highest scaffolding power ensures that contigs mapping to multiple orthologous proteins are scaffolded together by the ortholog that has the highest scaffolding power. After all guide proteins above a minimum scaffolding power have been chosen, the scaffolded contigs go through another scaffolding phase in which contigs with multiple gene models are used to merge the scaffolds together. The relative positions of contigs are clear when a single protein maps to them because of the linear structure of a gene. However, the loci of some proteins overlap and inferring the relative positions of these different contigs can be problematic. To deal with simple cases, directed graphs are constructed according to the relative mapping position of the contigs on the proteins. The order in which the contigs are traversed in these graphs determines the contig ordering of the scaffolds. After this scaffolding step, SWiPS takes all scaffolded contigs and uses genewise to build the final gene models.

### 2.5 Genome assembly data and guide protein sets

The *C.intestinalis* genome sequence, version 2.0 (Dehal *et al.*, 2002) was retrieved from Ensembl (release 63). 80× coverage Illumina 50 bp paired-end reads were simulated using simLibrary and simNGS (http://www.ebi.ac.uk/goldman-srv/simNGS/) with default parameters. *C.intestinalis* was chosen owing to our underlying interest in deuterostomes and for the pragmatic reasons that it is small and has not undergone a whole genome duplication. SOAPdenovo was used with default parameters to assemble the simulated *C.intestinalis* reads (Li *et al.*, 2010b). For linguistic convenience, we refer to all SOAPdenovo-generated sequences as contigs, even though these sequences include contigs that have been scaffolded together using the insert size distributions of paired-end sequence data.

Next-generation sequencing paired-end data from *D.melanogaster* DNA sequence were obtained from the short read archives (accession SRX021790; MacKay *et al.*, 2012), and were used to build an assembly (N50 = 1441 bp) with SOAPdenovo. The human (NA18507) genome assembly (N50 = 61 980 bp), built using SOAPdenovo, was obtained from http://yh.genomics.org.cn/download.jsp.

For the comparison with ESPRIT (Dessimoz *et al.*, 2011), we obtained the genomic assembly of *C.milii* from http://esharkgenome.imcb.a-star.edu.sg. This 1.4× assembly consists of 647 131 contigs (754 MB) with an N50 of 1464 bp.

All protein sets used were retrieved from Ensembl, except for *Strongylocentrotus purpuratus* and *Saccoglossus kowalevskii* (Genbank); and *Branchiostoma floridae*, *Drosophila grimshawi*, *Heliconius melpomene*, *Culex quinquefasciatus*, *Anopheles gambiae*, *Anopheles merus* and *Anopheles quadriannulatus* (Uniprot).

### 2.6 Estimating errors

All simulated contigs were mapped to the *C.intestinalis*, *D.melanogaster* or *H.sapiens* reference genome (all three from Ensembl) using blastn and assigned a reference scaffold, strand and position. SWiPS-predicted scaffolds were considered correct if their mapped contigs are ordered correctly (within 100 kb of each other as eukaryotic introns are rarely >100 kb; for human, we used 200 kb) across the reference scaffold on the same strand. Note that contigs are allowed to be intercalated between exon-containing scaffolds without affecting the assessed accuracy. We use two measures of accuracy: (i) the percentage of contig joins that are correct—local link correctness, and (ii) the percentage of scaffolds (with at least one contig link) for which all local contig links are correct.

## 3 RESULTS

### 3.1 Mapping ability with proteins from the same species

The extent to which contigs can be scaffolded by protein sequences will depend on the amount of protein-coding content in the genome, and the ability to assign contigs to particular protein-coding sequences. The latter will depend on the extent of similarity between the guide proteins and the genome under consideration. To understand the best possible outcome for the scaffolding process, we first tested SWiPS’s ability to assemble a genome using a protein set from the same species as the genome.

We created a *C.intestinalis* genome assembly of the kind that might be produced using a simple next-generation sequencing strategy, with low N50 (section 2.5), and applied SWiPS, using *C.intestinalis* proteins as guides for the scaffolding process.

Our algorithm improved the N50 of our simulated assembly from 3851 bp to 6245 (62.2% increase) and reduced the number of scaffolds from 108 424 to 94 680, with 4134 out of 4510 (91.66%) scaffolding to be entirely correct (it should be noted that the parameters used in this analysis were not optimized to take into account that the guide proteins were of the same species).

### 3.2 Use of orthologous protein sequences and dependence on read depth

As a more realistic test, we scaffolded simulated *C.intestinalis* assemblies using orthologous protein sequences from *Danio rerio*, *S.purpuratus*, *Gallus gallus*, *H.sapiens* and *S.kowalevskii*. To test the dependency on sequencing depth, we produced assemblies based on the *C.intestinalis* genome at 10×, 20×, 40× and 80× coverage (section 2.5).

**Fig. 4.**
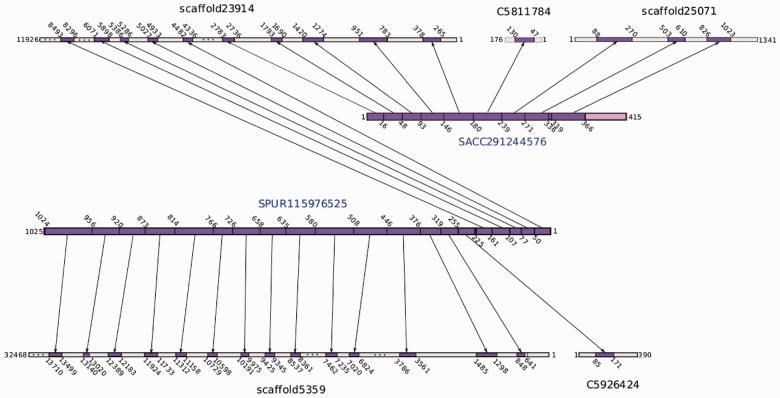
Example of five contigs scaffolded using SWiPS. Protein SACC29144576 links contigs ‘scaffold23914’, ‘C5811784’ and ‘scaffold25071’ together, whereas SPUR115976525 links contigs ‘scaffold23914’, ‘C5926424’ and ‘scaffold5359’ together. The two protein models allow SWiPS to scaffold all contigs together. Each arrow represents a region of homology (dark grey); the intron–exon structure (light grey and dark grey) of the orthologous proteins is clearly represented within each scaffold

Irrespective of coverage depth, SWiPS was able to produce a 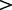
20% improvement in the N50 of the genome assembly, with a corresponding increase in the numbers of scaffolded contigs (Table 1). These numbers show that SWiPS appears to be useful for improving assembly quality for even low coverage (by next generation standards) datasets. Figure 4 shows an example of a scaffolded region, with two proteins, one from Sea Urchin and one from *Saccoglossus* providing evidence to link five contigs from the SOAPdenovo assembly.

**Table 1.** Breakdown of the scaffolding accuracy by assembly coverage

Assembly (coverage)	N50 improvements (bp)	CEGMA improvements (complete/partial genes)	Scaffold correctness	Local link correctness
*Ciona* 10×	1767–2101 (18.9%)	95–136 (41.2%)	88.3%	97.05%
		140–190 (35.7%)		
*Ciona* 20×	3714–4544 (22.3%)	134–166 (23.9%)	87.53%	97.42%
		175–204 (16.6%)		
*Ciona* 40×	3799–4790 (24.4%)	138–166 (20.3%)	85.65%	96.78%
		169–210 (24.3%)		
*Ciona* 80×	3851–4719 (22.5%)	139–162 (16.4%)	89.43%	97.60%
		171–210 (22.8%)		
*D.melanogaster*	1441–1601 (11.1%)	178–201 (12.9%)	98.9%	99.7%
		241–245 (1.7%)		
Human (NA18507)	61 980–73 255 (18.2%)	95–97 (2.1%)	85.3%^a^	96.0%^a^
		199–206 (3.5%)		
Elephant Shark	1464–1512 (3.3%)	7–22 (214.3%)	N/A	N/A
		49–107 (118.4%)		
				

^a^Since human genes are known to possess longer introns than general eukaryotic genes, we allowed 200 kb (instead of 100 kb) to assess the accuracy of SWiPS on the human assembly.

To test SWiPS on real next-generation data, we produced a *D.melanogaster* assembly from Illumina Genome Analyzer II sequence data. Using protein sequences from *D.grimshawi*, *H.melpomene*, *C.quinquefasciatus*, *Caenorhabditis elegans*, *A.gambiae*, *A.merus* and *A.quadriannulatus*, SWiPS produced an improvement of 11.1% on the assembly N50 and reduced the number of contigs from 130 312 to 123 309 (Table 1). We also tested SWiPS on a human assembly using protein sequences from *Canis familiaris* and *Mus musculus*. In this case, SWiPS produced an improvement of 18.2% on the assembly N50 and reduced the number of contigs from 314 877 to 306 882. We also found that, when allowing only 100 kb between scaffolded contigs (section 2.6), the assessed scaffold correctness was 70.6% and the local scaffold correctness was 90.3%. However, allowing 200 kb (500 kb) resulted in an assessed scaffold correctness of 85.3% (92.1%) and a local scaffold correctness of 96.0% (98.3%).

We also tested SWiPS on low coverage Sanger sequenced data, in the form of the 1.4× coverage genome of the elephant shark *Callorhinchus milli* (Venkatesh *et al.*, 2007). We obtained an improvement in N50 from 1464 bp (647 131 scaffolds) to 1506 bp (630 493 scaffolds), a 2.9% increase.

### 3.3 Comparison with other methods

To our knowledge, the only other program currently available to scaffold genomic contigs using protein sequences is ESPRIT (Dessimoz *et al.*, 2011). To directly compare SWiPS with ESPRIT, we applied the CEGMA pipeline to the *C.milii* reference genome. CEGMA uses a set of 248 core eukaryotic genes that are generally present in low copy number to assess the completeness and quality of a genome assembly, and can serve as complementary metric to the N50 length (Parra *et al.*, 2009).

In our hands, the CEGMA pipeline detected 7 complete and 47 partial genes when assessing the initial reference genome, while Dessimoz *et al.* reported 14 complete and 35 partial genes. This difference is likely due to our use of a more recent version of CEGMA. The overall similarity of the results, however, suggests a comparison of the performance of SWiPS and ESPRIT is valid.

To link *C.milii* contigs together with SWiPS, we used the same set of proteins used by ESPRIT, which consists of human, mouse, anole lizard, chicken, African clawed frog, zebrafish, medaka, *C.intestinalis* and *B.floridae* proteins. After linking 666 contig pairs with ESPRIT, Dessimoz *et al.* reported an increase from 14 to 16 complete genes and from 35 to 38 partial genes, i.e. a 14.3% and a 8.6% improvement, respectively. In comparison, SWiPS was able to merge 27 659 contigs into 9121 scaffolds, and increased the CEGMA gene space from 7 complete genes to 22 and 47 partial genes to 107, i.e. a 214.3% and a 118.4% improvement, respectively. Even using Dessimoz *et al.* higher baseline of 14 complete genes, the new assembly produced by SWiPS improved significantly the complete gene space when compared with ESPRIT (8 compared to 2, i.e. a 4-fold improvement).

## 4 DISCUSSION

We have shown that our method is able to use protein sequences to order and orient contigs into scaffolds, thus improving assembly contiguity and gene prediction, showing significant improvements over the only other available tool. Although the overall improvements in N50 found by SWiPS may appear modest, they should be understood with reference to the fact that the majority of most animal genomes is composed of non-coding sequence, and thus not amenable to scaffolding via protein coding sequences.

To confidently scaffold exon-containing regions on different contigs, we need to be sure that they come from the same gene. In the context of this problem, the most obvious way of doing this is by assuming that our guide protein comes from a gene that shares a 1:1 or many:1 orthologous relationship with the gene encoded by the exons. We thus need to test that each exonic region on a contig is orthologous to the guide protein. Rather than constructing a full phylogeny for each exon region, we maximize the overall similarity score of templates to exons for all proteins that match equivalent sets of regions. As the ability to discriminate orthologs from paralogs is fundamental to our method, we expect it to perform best for proteins that have no similar sequences in the genome, and less well where proteins come from gene families with multiple closely related members. Furthermore, the more closely related the template set of proteins is to the target genome, the less likely our method is to be confounded by gene duplication events that have occurred after the template and target lineages have diverged.

Although inference of orthology is most obvious via overall comparisons of sequence similarity, it is conceivable that other sources of information may be useful. For instance, conservation of exon/intron boundaries may help to discriminate between orthologs and paralogs in cases where orthologs share intron locations to the exclusion of paralogs. In practice, we were able to find few examples where this provided a significant improvement on our current results (data not shown).

For many applications, the primary interest of a genome sequence lies in its encoded proteins. Coupled with the difficulty of *de novo* genome assembly and gene prediction, this suggests that transcriptome sequencing may be a more useful strategy for generating an initial survey of genome content. Transcriptome sequencing, however, has major disadvantages compared with genome sequencing. Firstly, not all genes are expressed in all developmental stages and cell types, and for many taxa, it may be difficult to sample sufficient transcriptional libraries to obtain representation of all genes. Without this, it is difficult to make reliable inferences of the absence of particular genes. Secondly, genome sequence contains a wealth of information not encoded in the transcriptome, in the form of regulatory elements, synteny information, gene structures and so on. Preliminary genome sequence is thus likely to be of greater long-term usefulness than incomplete transcriptome data. Although genome assemblies produced from single libraries of Illumina data (i.e. with only one insert size) are likely to be very fragmentary (whatever the sequencing depth), protein-coding content is likely to be depleted in the repetitive sequence that causes assembly problems, at least at the exon level. By allowing the scaffolding of exons on different contigs, our method allows the maximum usefulness in terms of protein-coding content to be extracted from these preliminary assemblies.
